# MicroRNAs in clear cell renal cell carcinoma: biological functions and applications

**DOI:** 10.15586/jkcvhl.2015.40

**Published:** 2015-08-23

**Authors:** Gianluca Aguiari

**Affiliations:** Department of Biomedical and Surgical Specialty Sciences, Section of Biochemistry, Molecular Biology and Medical Genetics, University of Ferrara, Italy.

## Abstract

MicroRNAs (miRs) are small noncoding RNAs that govern many biological processes. They frequently acquire a gain or a loss of function in cancer and hence play a causative role in the development and progression of neoplasms. They could be used as biomarkers to improve our knowledge on diagnosis, prognosis and drug resistance, and to attempt therapeutic approaches in several types of cancer including clear cell renal cell carcinoma (ccRCC). ccRCC is the most predominant subtype of RCC that accounts for about 90% of all renal cancers. Since ccRCC is generally asymptomatic until very late, it is difficult to diagnose early. Moreover, in the absence of preventive treatments for metastatic ccRCC after surgical resection of the primary cancer, predictive prognostic biomarkers are needed in order to achieve appropriate therapies. Herein the role of miRs in the biology of ccRCC and the potential applications of these molecules are discussed. Moreover, future applications in the diagnostic and prognostic field, as well as their impact on drug response and therapeutic targets are also explored. Their use in clinical practice as molecular biomarkers alone, or in combination with other biological markers could accelerate progress, help design personalized therapies, limit side effects, and improve quality of life of ccRCC patients.

## Introduction

Renal cell carcinoma (RCC) accounts for 2–3% of all cancers and is the most common kidney malignancy with the highest mortality rate of urinary cancers. Lifestyle factors including smoking, obesity and hypertension are considered etiological risk factors for this tumor (1–3). RCC is mainly classified into three major subtypes: clear cell (ccRCC), papillary (pRCC) and chromophobe (chRCC). The ccRCC subtype is the most common accounting for 80–90% of RCC cases (3). Generally, ccRCC arises from the epithelial cells of the proximal convoluted renal tubule and is histologically characterized by clear cells separated by hypervascular thin fibrous septae (4–5). This subtype of renal cancer commonly metastasizes to the bones, lungs, brain and liver through the invasion of the vena cava (6). Currently, partial or radical nephrectomy is the only curative treatment for localized tumors with high quality of life outcomes (3). In comparison, for patients with metastatic ccRCC, nephrectomy is mostly palliative and systemic treatment with pharmacological therapy is necessary. Unfortunately, ccRCC is unresponsive to traditional chemotherapies, highly resistant to radiation, and lacks the hallmark genetic features of solid tumors, such as mutations in KRAS and TP53 genes (7).

Recent advances in molecular biology have led to the identification of a plethora of gene mutations associated with the development and progression of ccRCC. It is well known that the von Hippel-Lindau (VHL) gene, localized on chromosome 3p, is frequently inactivated either by mutation or methylation in over 80% of ccRCC patients. The VHL protein complexes with other proteins, and functions as an E3 ubiquitin ligase. This leads to degradation of the á subunit of hypoxia-inducible transcription factors (HIF1 and HIF2) via proteasome activation (8–9). The loss of function of VHL prevents the degradation of HIFá proteins resulting in the increased expression of angiogenic factors including vascular endothelial growth factor (VEGF) and platelet-derived growth factor B chain (PDGF-B) that contribute to growth and expansion of tumor (10). Mutations in mammalian target of rapamycin (mTOR), TSC1, PIK3CA, and PTEN genes in approximately 20% of ccRCC have also been observed. These molecular lesions cause an aberrant mTORC1 pathway activation that could play a relevant role in the neoplastic transformation of kidney cells (8). The discovery of several altered signaling pathways associated with ccRCC has opened new opportunities and strategies in the treatment of ccRCC, especially the targeted inhibition of molecules involved in VEGF and mTOR pathways (11–12). However, complete remission or long-term beneficial responses with tyrosine kinase or mTOR inhibitors are rare (12). Moreover, these pharmacological approaches are expensive and show significant adverse effects that worsen the quality of life of ccRCC patients. Thus, cost-effective predictive biomarkers that are able to improve clinical management of ccRCC patients are needed. In this regard, an attractive solution could be offered by microRNAs (miRs) that are involved in the development of many cancers. Herein we discuss the role of miRs in RCC biology and their possible applications as biomarkers for ccRCC.

## Biogenesis and function of microRNAs

MiRs are short non-coding single stranded RNAs of 20–22 nucleotides in length that regulate gene expression at the post-transcriptional level. They act by inhibiting the translation of mRNA primarily by pairing with the 3′-untranslated region (UTR) of their complementary mRNAs (13). MiRs are transcribed by RNA polymerase II or RNA polymerase III into a long primary nuclear miR (primiRNA) that is cleaved to 70–80 bp long pre-miRNA by the Drosha/DGCR8 complex and exported into the cytoplasm via exportin-5/RanGTP (14–15). Pre-miRNAs are then digested to double-stranded mature miRs by the RNAse III Dicer, and separated in two strands: the miRNA-guide strand and the miRNA-passenger strand. The miRNA-guide strand is loaded into the RNA-induced silencing complex (RISC) and driven to the target mRNA, while the miRNA-passenger strand is generally degraded. MiRNA gene silencing may be performed through degradation via complete pairing, where the mRNA target is degraded; or incomplete pairing, with the inhibition of translation or the decay of mRNA into cytoplasmic foci termed P bodies (14). MiRs regulate several cellular processes such as apoptosis, proliferation, hematopoiesis and angiogenesis (13). Pertinent to tumors, they can act either as pro- or anti-oncogenic molecules, thereby having a dual role in oncogenesis. They can act either as tumor suppressors by targeting oncoproteins or as tumor promoters by downregulating the expression of tumor suppressor proteins (16–17). Moreover, a large number of miRs have been identified to regulate cellular metabolism that is often altered in tumor cells contributing to development and progression of cancer (18).

## MicroRNAs affect biological processes in ccRCC

**Figure 1:**
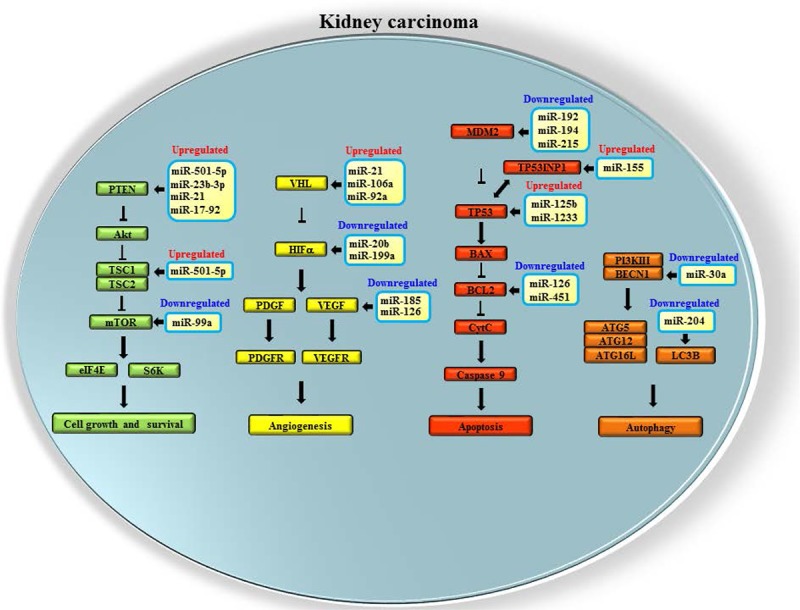
Dysregulated microRNAs affecting cell growth, angiogenesis, apoptosis and autophagy in kidney cancer. In the sketch, microRNAs targeting genes involved in mTOR (green), VHL/HIF-α (yellow) and p53 (red) pathways as well as in autophagic process (orange) are shown.

It is well known that miRs are involved in the pathophysiology of a variety of kidney diseases including diabetic nephropathy, polycystic kidney disease, Wilms tumor and kidney cancer (13). This section focuses on the role of miRs in kidney cancer with emphasis on cell growth, angiogenesis, apoptosis and autophagy (**Figure 1; Table 1**).

**Table 1:** MicroRNAs dysregulated in renal cancer

**MicroRNAs**	**Target genes**	**Expression**	**Role**	**Applications**	**Ref.**
**miR-501-5p, miR-23b-3p**	*TSC1, PTEN, MCU*	Up	Cell growth, metastasis	Prognosis	2, 28
**miR-21, 106a, 92a**	*VHL, PTEN, PDC4*	Up	Cell growth, migration	Prognosis	19–20, 26–27, 52
**miR-210, 155**	*ISCU1/2, TP53INP1*	Up	Metastasis, cell growth	Diagnosis/prognosis	20, 24, 44–45, 49–50
**miR-20b, 199a**	*HIF-α*	Down	Anti-angiogenesis	-	25
**17–92 cluster**	*PTEN, E2Fs*	Up	Cell growth, invasion	Prognosis	20–22, 24
**miR-28-5p**	*Mad2*	Up	Chromosomal instability	-	23
**miR-192, 194, 215**	*ZEB2, MDM2, TYMS*	Down	Cell differentiation	Prognosis	29
**miR-584**	*ROCK1*	Down	Cell cycle arrest	-	30
**miR-135a**	*cMYC*	Down	Cell cycle arrest	-	31
**miR-205**	*SFKs*	Down	Cell cycle arrest, apoptosis	-	32
**miR-708**	*Survivin, ZEB2, BMI1*	Down	Apoptosis	-	33
**miR-1826**	*CTNNB1, MEK1*	Down	Cell cycle arrest, apoptosis	-	34
**miR-30d, 185**	*MTDH, VEGFA*	Down	Apoptosis	-	35–36
**miR-204**	*LC3B*	Down	Autophagy	-	37–38
**miR-30a**	*BECN1*	Down	Inhibition of autophagy	-	39
**miR-203, 424**	*FGF2, PDCD4*	Up	Cell proliferation	Diagnosis	41
**miR-195, 15b**	*Raf-1, CCND1*	Up	Cell cycle arrest	Diagnosis	42
**miR-122-5p**	*HIF1- α vimentin*	Up	Inhibition of cell invasion	Diagnosis	43
**miR-378, 451**	*TOB2, BCL2*	Up/down	Apoptosis, cell cycle	Early diagnosis	46
**miR-1233**	*BLCAP, TP53*	Up	Inhibition of apoptosis	Early diagnosis	47
**miR-193a, 362, 572, 28, 378**	*MAPK, NFkB, Mad2*	Up/down	Cell growth and invasion	Early diagnosis	48
**miR-126**	*VEGFA, BCL2*	Down	Cell migration, apoptosis	Prognosis	51–52
**miR-125b**	*E2F3, TP53*	Up	Cell growth and migration	Prognosis	53
**miR-30c**	*Snail1/slug, PTP4A1*	Down	Inhibition of cell migration	Prognosis	55
**miR-200c**	*CYP1B1, HO-1*	Down	Inhibition of cell invasion	Drug response	58–59
**miR-942**	*BRCA pathway*	Up	Angiogenesis, metastasis	Drug response	60
**miR-141**	*ZEB2*	Down	Cell differentiation	Drug response	61
**miR-200**	*IL-8, CXCL1*	Down	Anti-angiogenesis	Therapy	63
**miR-99a**	*mTOR*	Down	Cell cycle arrest	Therapy	64

### Cell growth and migration

As observed in other tumors, miRs may contribute to development of RCC by modulating the expression of genes involved in this cancer. For example, VHL is targeted by miR-106a, miR-92a and miR-21, which have been found upregulated in renal carcinoma tissues (19–20). Moreover, VHL loss of function can activate the expression of different miRs including the miR-17–92 cluster, also known as oncomiR-1. These miRs act as oncogenic agents targeting PTEN and E2Fs tumor suppressor genes (21–22). Furthermore, VHL deletion/mutation also causes the enhancing of miR-28-5p expression that in turn induces the reduction of Mad2 protein levels, thereby promoting checkpoint weakness and chromosomal instability in ccRCC cells and tissues (23). In addition, the impaired VHL signaling causes the activation of HIF pathway, which leads to the upregulation of miR-210 and miR-155 which have been identified as having oncogenic properties in ccRCC (19–20, 24). Conversely, hypoxia can lead to the downregulation of miRs that target the HIF1á transcripts. MiR-20b and miR-199a are repressed under conditions of hypoxia (25). Furthermore, hypoxia may reduce miRs that target VEGF and promote angiogenesis (25). Besides VHL gene, miR-21 may degrade PTEN mRNA leading to the activation of Akt and mTOR kinases which contribute to growth and migration of kidney cancer cells (26). In ACHN and 786-0 cells, miR-21 induced migration and invasion through the modulation of the pro-apoptotic protein PDCD4 (19, 27).

In addition, it also promoted tumor metastasis by negatively regulating the tumor suppressor gene tropomyosin-1 (19). Other miRs targeting PTEN and mTOR mRNAs such as miR-501-5p and miR-23b-3p show oncogenic features in different ccRCC cell lines (2, 28). In ccRCC tissue of patients that developed distant metastases, a significant increase of miR-501-5p expression was observed (2). In KJ29 and Caki-2 ccRCC cells, miR-501-5p stimulated cell proliferation via mTOR-mediated MDM2 upregulation. MiR-23b-3p was upregulated in A-498 and Caki-2 ccRCC cell lines and its downregulation induced apoptosis and reduced invasion (28). In patients, an increased expression correlated with lower survival rate (28).

### Cell differentiation and apoptosis

The downregulation of miRs targeting specific oncogenes may contribute to the development and progression of RCC. In this regard, miR-192, miR-194 and miR-215 are highly expressed in normal kidney, but they are significantly downregulated in both primary and metastatic ccRCC. These miRs target ZEB2, MDM2 and TYMS mRNAs that are associated with cancer development and metastasis. The restoration of these miRs causes a reduction of cell migration and invasion in RCC cell lines (29). Moreover, miR-584, which is downregulated in ccRCC tissues, functions as a tumor suppressor miR that inhibits cell motility by the negative regulation of ROCK-1 oncogene expression (30). Similarly, miR-135a was found significantly reduced in renal cancer tissues and its restoration in both Caki-2 and A498 ccRCC cell lines caused cell cycle arrest in G0/G1 phase. It also targeted the c-MYC oncogene which was upregulated in ccRCC tissues (31). Cell cycle arrest may be also induced by miR-205 restoration that leads to inhibition of Src family kinases (SFKs) expression, stimulating apoptosis and inhibiting cell proliferation, migration, and colony formation in renal cancer cells. This miR is significantly suppressed in renal cancer cell lines and tumor tissues (32).

Pro-apoptotic miRs are often downregulated in ccRCC. For instance, miR-708, which exerts pro-apoptotic effects in ccRCC cells and tissues by targeting survivin, ZEB2 and BMI1 genes that are involved in cell survival, adhesion, invasion, and metastasis, has been found widely reduced in human RCC tissues. The restoration of miR-708 dramatically increases apoptosis in ccRCC cell lines. Moreover, the intra-tumoral delivery of this miR stimulates the regression of tumors in murine xenograft models of human RCC (33). Similarly, miR-1826 that is significantly downregulated in renal cancer tissues acts as tumor suppressor agent promoting apoptosis and cell cycle arrest by knockdown of CTNNB1 (beta-catenin) and MEK1 genes in VHL-inactivated ccRCC cells (34). The miRs miR-30d and miR-185 also may function as onco-suppressors in ccRCC cells inducing apoptosis through the inhibition of the oncoprotein metadherin (MTDH) and VEGF A (VEGFA) expression (35–36).

### Autophagy

Autophagy is an important mechanism that plays both pro- and anti-oncogenic roles. In kidney cancer, the pro- or anti-oncogenic role of autophagy is mediated by two different forms of autophagic protein LC3, LC3B and C, which are regulated by the VHL protein. The expression of LC3B supports tumor formation and progression in renal cancer cells with VHL loss of function, whilst the expression of LC3C shows tumor suppressor activity (37). A functional VHL protein inhibits LC3B-mediated autophagy through the stimulation of miR-204, which directly targets LC3B mRNA reducing its translation. In ccRCC cells and metastatic tissues, most of which are characterized by early inactivation of the VHL gene, a reduction of miR-204 expression as well as an increase of LC3B protein levels have been reported. Thus, in kidney cells, miR-204, in a network involving VHL, functions as a tumor suppressor miR that inhibits pro-oncogenic autophagy by targeting LC3B mRNA (37–38). The expression of miR-30a may also affect autophagy in kidney cancer. It acts as an onco-suppressing agent and inhibits autophagy by targeting the BECN1 gene that codes for beclin-1, a protein crucial for the formation of the autophagosome. This miR is significantly reduced in both human RCC tissues and cell lines, and its restoration not only causes the inhibition of autophagy by the reduction of beclin-1 protein but also enhances sorafenib-induced cytotoxicity in different ccRCC cell lines (39). Autophagy may represent an attractive target for the design of new therapeutic approaches in chemo-resistant metastatic renal cancer by using specific drugs for the inhibition of pro-oncogenic autophagy. However, further studies are needed to evaluate the pro- or anti-oncogenic role of autophagy in renal cancer.

## MicroRNAs and their applications in ccRCC

**Figure 2:**
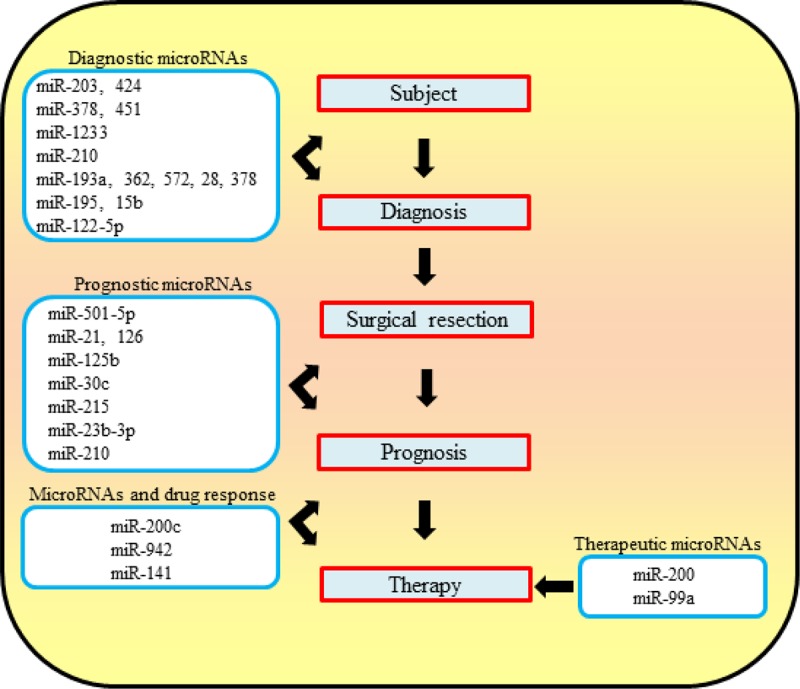
Flow chart of possible future management of RCC patients by using microRNAs. MicroRNAs with diagnostic, prognostic and therapeutic properties along with drug response are indicated. Prognostic miRs should allow the identification of ccRCC patients that need specific therapeutic treatments from those which do not need pharmacological therapy.

In RCC patients after surgical resection no effective preventive adjuvant therapy is available. The likelihood of relapse or metastases in high grade ccRCC is frequent (40). Moreover, the lack of specific prognostic biomarkers prevents the development of specific therapy. Thus, the discovery of new biomarkers for prognosis of ccRCC could be useful for the clinical management of these cancers (2). In this regard, miRs that are involved in carcinogenesis could be considered as predictive biomarkers for kidney cancer. The stable nature of these molecules and their relative ease of detection and recovery in biological fluids make them optimal candidates as molecular biomarkers. In particular, they could be used as diagnostic/prognostic tools and for the design of new therapeutic strategies (**Figure 2**).

### MicroRNAs for the identification of kidney cancer subtypes

Immunohistochemical markers do not allow the accurate identification of tumor subtypes, especially in the setting of uncommon morphology and small biopsies. However, miRs that are well established as regulators of tumorigenesis could be utilized as potential biomarkers alone or in combination with current markers in order to improve the classification of kidney cancer. In this regard, miR-424 and miR-203 that are overexpressed in ccRCC but not in pRCC could be used to distinguish ccRCC from pRCC (41). Also miR-195 and miR15b are upregulated in ccRCC compared with pRCC, chRCC and oncocytoma (42). The expression of miR-126-3p is higher in ccRCC than in pRCC tissues, while miR-122-5p, which is highly expressed in liver, was specifically expressed in ccRCC but not in the other subtypes of RCC (43). The differential expression of 24 miRs in different kidney cancer subtypes was reported to distinguish all 4 common subtypes of kidney tumors with an overall accuracy of 95% (43). Therefore, these miRs could be considered as diagnostic biomarkers in order to improve the classification of renal cell carcinoma.

### Circulating microRNAs for early diagnosis of ccRCC

Circulating miRs could serve as non-invasive biomarkers for the early diagnosis of kidney carcinoma. In this regard, miR-210 that is strongly upregulated in ccRCC tissue, is also abundantly found in the serum of ccRCC patients compared with healthy controls. These findings suggest that miR-210 could be used as biomarker for early diagnosis of ccRCC (19,44–45). Other studies report that levels of miR-378 are increased, while those of miR-451 are decreased in serum of RCC patients compared with healthy controls. The detection of miR-378 and miR-451 in serum allows the identification of patients with RCC with a sensitivity of 81% and a specificity of 83%, making the combination of these miRs a possible early diagnostic biomarker for RCC (46). Also the circulating miR-1233 could also serve as a potential diagnostic biomarker for this cancer (47). Recently, it has been reported that serum levels of miR-193a-3p, miR-362 and miR-572 are significantly increased whereas those of miR-28-5p and miR-378 are decreased in patients with RCC compared with the healthy controls. The combined use of these 5 miRs could serve as a biomarker panel for the identification of early stage renal cancer with a sensitivity of 80% and a specificity of 71% (48). However, further studies are needed to validate candidate circulating miRs as biomarkers for early diagnosis of renal cancer by using large multicentre-cohorts of patients.

### MicroRNAs for ccRCC prognosis

Apart from early diagnosis, miRs could also serve as prognostic markers. In this regard, a high level of miR-210 was associated with a good clinico-pathological outcome in ccRCC patients (49), however, other studies have associated a high miR-210 with a higher chance of disease recurrence and shorter overall survival (24, 50). This discrepancy suggests that further studies to define the role of miR-210 in ccRCC are needed. Lower levels of miR-215 (29) and miR-126 (51) are associated with a worse outcome in RCC patient and a higher expression of miR-126 is associated with significantly prolonged disease-free survival and overall survival (51). Furthermore, an increased miR-501-5p, miR-23b-3p (2, 28), miR-21 (52), miR-125b (53) and a decreased miR-126 (52) are indicators of worse outcome in metastatic patients. Other studies report that the high miR-21/10b ratio is associated with a poor prognosis in non-metastatic ccRCC patients (54). Recently, it has been published that the differential expression of miR-30c, miR-451 and miR-126 are associated with overall survival in ccRCC patients (55). A miR signature that includes the upregulation of miR-21-5p, 142-3p, let-7g-5p, let- 7i-5p and 424-5p, as well as the downregulation of miR-204-5p is associated with high stage, grade and progression of ccRCC (56). Another miRNA signature consisting of 22 miRNAs that are significantly linked to patient survival in ccRCC was recently identified (57). Finally, it has also been reported that the expression some miRs may predict site of metastasis. For example, the decreased expression of miR-10b is associated with brain metastasis, while the upregulation of miR-199b and the downregulation of miR-615 are linked to lung metastasis in patients with advanced ccRCC (55).

### MicroRNAs and drug resistance

In addition to their role in tumor progression, miRs could also affect response to therapy. The downregulation of miR-200c in RCC causes increased expression of the CYP1B1 gene resulting in pharmacological resistance to docetaxel treatment (58). Moreover, the upregulation of miR-200c enhanced the sensitivity of ccRCC cells to sorafenib or imatinib, inhibiting cell proliferation by targeting heme oxygenase-1 (HO-1) mRNA (59). The upregulation of miR-942, miR-133a, miR-628-5p, and miR-484 was associated with sunitinib resistance in metastatic RCC patients. In particular, the increased expression of miR-942 alone could accurately predict sunitinib resistance that occurs by MMP-9 upregulation and VEGF secretion (60). Other studies show that miR-141 downregulation drives epithelial-to-mesenchymal transition (EMT) in ccRCC and is associated with an unfavorable response to sunitinib treatment. In vitro reintroduction of miR-141 reversed EMT and decreased cell viability in hypoxic conditions (61). To date, about 28 miRNAs related to poor response and 23 associated with prolonged response to sunitinib treatment have been identified (62).

### MicroRNAs for gene therapy

As previously discussed, miRs may act as oncogenes or onco-suppressors, contributing to development, growth and metastasis of RCC. Therefore, the restoring of normal levels of onco-suppressor miRs or the degradation of onco-miRs by using specific anti-miR sequences could be an attractive therapeutic strategy for the treatment of metastatic ccRCC. In this regard, the downregulation of the miR-200 family in different tumors is associated with cell migration, invasion, and metastasis by the increased expression of IL-8 and CXCL1 genes that promote angiogenesis. The delivery of miR-200 members into the tumor endothelium of orthotopic cancer models by using specific nanoparticle resulted in marked reduction of metastasis and angiogenesis (63). The downregulation of miR-99a in RCC tissues was associated with distant metastases and poor prognosis in RCC patients through the activation of the mTOR pathway. The restoration of miR-99a levels in RCC cells inhibited cell growth, migration and invasion. Moreover, intra-tumoral delivery of miR-99a reduced tumor growth in murine xenograft models of human RCC (64). The development of specific miR-targeted therapies, such as the reintroduction of onco-suppressor miRNAs or the inhibition of onco-miRs in RCC cells, could have significant translational implications such as improvement of the clinical picture, management and quality of life of RCC patients. However, there are several obstacles to deliver miRs/anti-miRs into the tumor cell including low cellular uptake, endosomal escape, immunogenicity, degradation in the bloodstream, and rapid renal clearance. Moreover, for a successful miR-based therapy, specific delivery of miR molecules to target the organ is important. There is an urgent need for developing efficient transfection methods as well as target-specific delivery systems to realize the full therapeutic potential of miRs (65).

## Conclusions

This review gives an overview of the impact of miRs in renal tumorigenesis, their potential role as biomarkers and therapeutic targets. MiRs show a relative versatility of use, however they may also show some practical disadvantages largely because of problems associated with accurate quantification in biological fluids. In fact, miRNA analysis in paraffin embedded tissues as well as in serum or in other biological fluids may be complicated by the absence of specific methods of standardization and suitable cut-off values. Furthermore, miR expression could be affected by genetic factors, environment, lifestyle and by sample collection methods. Despite these potential limitations, a large number of studies showing miRs as predictive biomarkers in different cancer types are emerging. Further studies using large independent cohorts of ccRCC patients are needed to validate miRs as biomarkers that are useful in clinical practice. The combination of miRs and other renal cancer biomarkers such as VEGF and VEGF-related proteins, cytokines and lactate dehydrogenase (66) could improve the reliability of these putative biomarkers. Therefore, miRs could become a powerful tool for clinical management of high grade and metastatic ccRCC patients, opening new horizons to achieve personalized medicine.
